# Inhibition of Ceramide Synthesis Reduces α-Synuclein Proteinopathy in a Cellular Model of Parkinson’s Disease

**DOI:** 10.3390/ijms22126469

**Published:** 2021-06-16

**Authors:** Alessandra Mingione, Francesca Pivari, Nicoletta Plotegher, Michele Dei Cas, Aida Zulueta, Tommaso Bocci, Marco Trinchera, Elisabetta Albi, Vittorio Maglione, Anna Caretti, Luigi Bubacco, Rita Paroni, Daniele Bottai, Riccardo Ghidoni, Paola Signorelli

**Affiliations:** 1 Biochemistry and Molecular Biology Laboratory, Department of Health Science, University of Milan, 20142 Milan, Italy; francesca.pivari@unimi.it (F.P.); aida.zulueta@guest.unimi.it (A.Z.); anna.caretti@unimi.it (A.C.); 2 “Aldo Ravelli” Center for Neurotechnology and Experimental Brain Therapeutics, University of Milan, 20142 Milan, Italy; tommaso.bocci@unimi.it (T.B.); marco.trinchera@uninsubria.it (M.T.); daniele.bottai@unimi.it (D.B.); riccardo.ghidoni@unimi.it (R.G.); 3 Department of Biology, University of Padova, 35121 Padova, Italy; nicoletta.plotegher@unipd.it (N.P.); luigi.bubacco@unipd.it (L.B.); 4 Laboratory of Clinical Biochemistry and Mass Spectrometry, Department of Health Sciences, University of Milan, 20142 Milan, Italy; michele.deicas@unimi.it (M.D.C.); rita.paroni@unimi.it (R.P.); 5 Department of Medicine and Surgery, University of Insubria, 21100 Varese, Italy; 6 Department of Pharmaceutical Sciences, University of Perugia, 06123 Perugia, Italy; elisabetta.albi@unipg.it; 7 IRCCS Neuromed, 86077 Pozzilli, Italy; vittorio.maglione@neuromed.it; 8 Department of Health Sciences, University of Milan, 20142 Milan, Italy

**Keywords:** Parkinson’s disease, α-synuclein, sphingolipids, ceramide, myriocin, autophagy, oxidative stress

## Abstract

Parkinson’s disease (PD) is a proteinopathy associated with the aggregation of α-synuclein and the formation of lipid–protein cellular inclusions, named Lewy bodies (LBs). LB formation results in impaired neurotransmitter release and uptake, which involve membrane traffic and require lipid synthesis and metabolism. Lipids, particularly ceramides, are accumulated in postmortem PD brains and altered in the plasma of PD patients. Autophagy is impaired in PD, reducing the ability of neurons to clear protein aggregates, thus worsening stress conditions and inducing neuronal death. The inhibition of ceramide synthesis by myriocin (Myr) in SH-SY5Y neuronal cells treated with preformed α-synuclein fibrils reduced intracellular aggregates, favoring their sequestration into lysosomes. This was associated with TFEB activation, increased expression of TFEB and LAMP2, and the cytosolic accumulation of LC3II, indicating that Myr promotes autophagy. Myr significantly reduces the fibril-related production of inflammatory mediators and lipid peroxidation and activates NRF2, which is downregulated in PD. Finally, Myr enhances the expression of genes that control neurotransmitter transport (SNARE complex, VMAT2, and DAT), whose progressive deficiency occurs in PD neurodegeneration. The present study suggests that counteracting the accumulation of inflammatory lipids could represent a possible therapeutic strategy for PD.

## 1. Introduction

Parkinson’s disease (PD) is a progressive form of neurodegeneration that can be either sporadic or caused by various genetic mutations, affecting 1% of people over the age of 60 and up to 4% of those over 85 worldwide [1]. PD is characterized by tremors, rigidity, an impairment of movements, gait and balance, depression, and, at later stages, dementia [1]. The postmortem brains of PD patients show lipid and protein aggregates named Lewy bodies (LBs). LBs are characterized by the presence of the lipid-binding protein α-synuclein (α-syn) and are associated with the loss of dopaminergic neurons, mainly in the substantia nigra pars compacta [2,3].

The impairment of cellular traffic is associated with Parkinson’s disease. SNCA (synuclein alpha) gene mutations, duplications, and triplications cause autosomal forms of PD, inducing presynaptic α-syn aggregation, the disruption of ER and Golgi trafficking, and the impairment of lysosomal function [4,5]. Among the genetic mutations associated with familial PD or those responsible for increasing the susceptibility to sporadic PD are a number that are involved in cellular traffic and lysosomal activities (VPS35, LRRK2, ATP6VOA1, CTS D, and CTS B) and in lipid metabolism (PLA2G6, SYNJ1, GALC, GBA, SMPD1, PANK2, SREBF1, DGKQ, and ASAH1) [4,5,6,7,8]. Such mutations cause proteinopathies, which lead to dysfunctions in mitochondrial activity, retromer function, and the sorting machinery for lysosomal degradation [9,10,11], as well as dysfunction of the SNARE complex, insuring the release of neurotransmitters [12,13]. All of the above-reported activities involve membrane traffic and require lipid synthesis and metabolism [14].

Proteinopathy-induced stress is alleviated by the proteasome system, in addition to autophagy. Both the proteasome system and autophagy are inefficient or impaired in PD, worsening stress conditions and inducing neuronal death [15,16,17]. Autophagy requires lipid metabolism for the formation of autophagosome membranes, targeting materials for lysosomal degradation. Recent evidence suggests that lipids can be involved in amplifying and accelerating the degenerative process in PD in dopaminergic neurons [18,19]. Lipid metabolism is controlled by transcription factor EB (TFEB), the master regulator of the stress response. TFEB promotes lysosomal biogenesis, autophagy induction, and lipid oxidation, favoring energy supply and anti-inflammatory and antioxidant responses [20,21]. TFEB interacts with α-synuclein, which is able to sequester TFEB in the cytosol in the dopaminergic neurons of PD patients, thus preventing its nuclear translocation and activity [16,17,22]. Similarly, the oxidative stress responder NRF2 is defective in PD, as observed in postmortem brains [23], and its pharmacological activation is proposed for PD therapy [24,25]. Given the crucial role of lipids in neuronal and synaptic development and in their functions [26], the defective lysosomal catabolism of lipids and their accrual are known to cause degeneration in the nervous system [7,27,28]. Mutations in the glucocerebrosidase gene (GBA) associated with reduced GBA enzymatic activity are the most common genetic risk factors for PD [29,30,31]. The peroxidation of accumulated lipids is, per se, an additive cause of α-syn aggregation [8,32,33,34]. In GBA-associated PD, but also in other pathogenetic mutations and in the idiopathic disease, the changes in lipid metabolism correlate with the severity of both motor and non-motor features [31,35]. PD mutations are specifically associated with the alteration of sphingolipid homeostasis. An increase in the sphingolipid ceramide is specifically involved in LB formation and PD progression [5,36,37]. The inhibition of de novo ceramide synthesis by myriocin (Myr, an inhibitor of serine palmitoyltransferase) reduced seizures and paralysis upon mechanical stimulation (bang sensitivity) in a Drosophila model of retromer dysfunction induced by PLAa2G6 [10]. 

Notably, lipid alteration has been reported in PD patients. Although the data in the literature are somewhat conflicting, there is an overall agreement on altered lipid metabolism in PD. The plasma of PD patients shows variable alteration of glycerophospholipids [7,38] and ceramides. Furthermore, hexosylceramides were shown to be significantly higher [39], correlating with psychiatric complications [40,41]. Moreover, an increase in C16:0 acyl chain ceramides was found in PD plasma [42]. Cholesterol and sphingomyelin are major components of myelin. Cholesterol accumulation is associated with the increased formation of intracellular lipid–protein aggregates [42], and oxysterols are increased in the PD brain [38]. Sphingomyelins and ceramides are increased in postmortem PD brains compared to those of healthy subjects [8,38,43], and it is notable that there is a marked increase in the transcription of the enzymes responsible for de novo sphingolipid synthesis [38]. We have previously shown that the inhibition of de novo sphingolipid synthesis by Myr administration to mice prevents an increase in ceramide levels and neuronal loss in a mouse model of retinitis pigmentosa [44]. Myr exerts an overall effect on cellular energy metabolism via the promotion of lipid consumption and ATP production by triggering autophagy and reducing inflammation and the loss of cell function [8,45]. In the present study, we investigated the effect of Myr treatment on a cellular model of PD with the aim of regulating lipid metabolism as a possible therapeutic strategy in restoring neurotransmitter traffic and neuronal activity in PD.

## 2. Results

### 2.1. Myriocin Reduces the Amount of Intracellular α-Syn in SH-SY5Y Cells Treated with Preformed Fibrils

**Figure 1:**
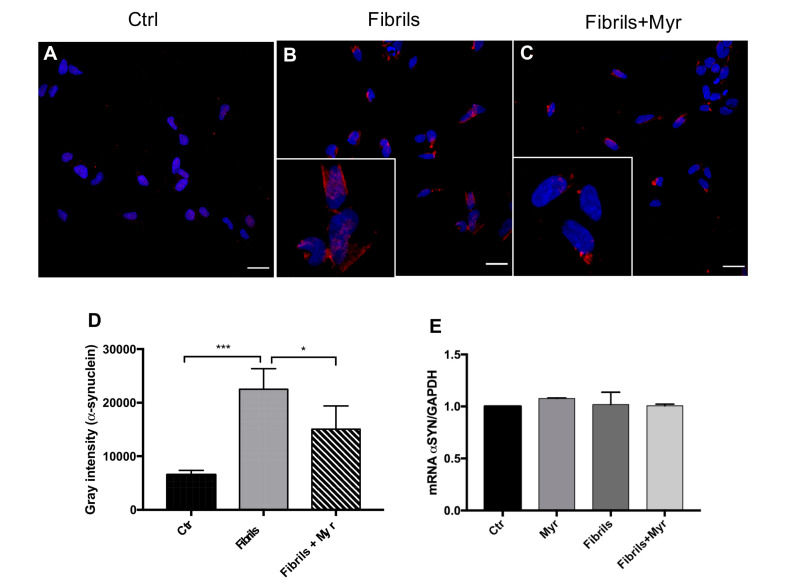
Myr reduces α-syn in SH-SY5Y cells. (**A**–**C**) SH-SY5Y cells were pretreated with or without 50 μM Myr and then exposed to 0.5 μM α-syn fibrils for 24 h. Immunofluorescent staining of α-syn (red) and nuclear staining with DAPI (blue) are shown. (**D**) Summary of results demonstrating the mean intensity per cell measured as grayscale intensity. Scale bars represent 20 μm. (**E**) α-syn gene expression measured by qRT-PCR and represented as fold change compared to control cells (Ctr). All data, derived from three independent experiments, are expressed as mean ± SEM (* *p* < 0.05; *** *p* < 0.001); one-way ANOVA test followed by Bonferroni correction was used for all data.

The preformed α-syn fibrils (hereinafter referred to as fibrils) were exogenously added to cell cultures of SH-SY5Y to mimic LB formation in PD. As expected, immunostaining with an α-syn antibody showed a low basal expression of endogenous α-syn (Figure 1A), whereas in fibril-treated cells, the staining was more diffused in the cytosol and distributed in the perinuclear region (Figure 1B). 

**Figure 2:**
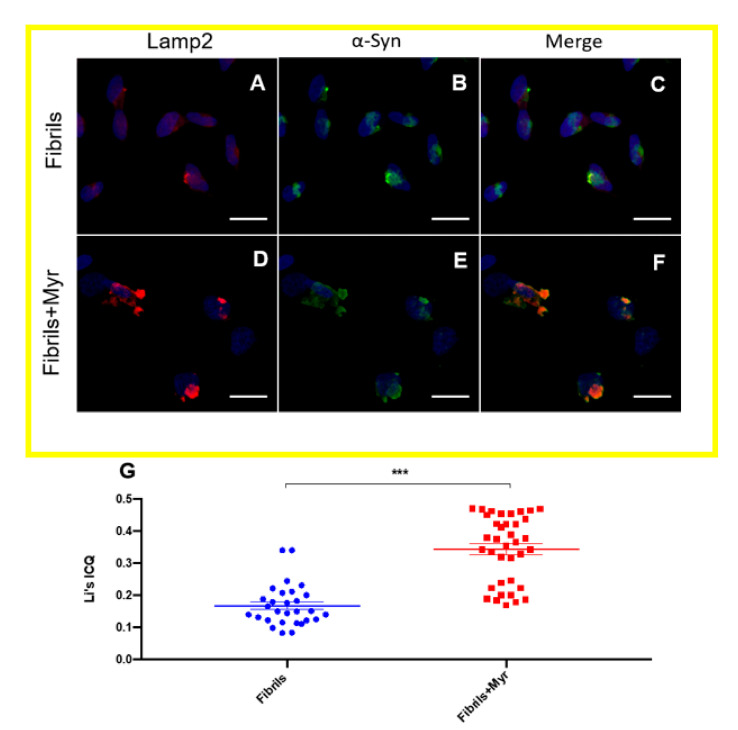
Myr induces co-localization of α-syn inclusions with the lysosomal marker. Confocal immunofluorescent images of SH-SY5Y cell line treated with 0.5 μM α-syn fibrils for 24 h with or without 50 μM Myr pretreatment. Confocal images were obtained after co-immunostaining of Lamp2 antibody (red, **A**,**D**) and α-syn antibody (green, **B**,**E**); co-localization in merge images is shown in yellow (**C**,**F**); DAPI (blue), nuclear counterstaining. Scale bars represent 20 μm. (**G**) Graph of quantification of co-localizing fluorophores’ intensity (Li’s ICQ: intensity correlation quotient). Three independent experiments were performed; the cells corresponding to the dot number were randomly selected among 4 fields of each group. Data are presented as the intensity correlation of single cells (*** *p* <0.001); unpaired Student’s *t*-test was used.

In order to evaluate if sphingolipid synthesis inhibition can reduce the stress caused by α-syn intracellular accumulation, SH-SY5Y cells were pretreated with Myr at 50 μM for 2 h before the addition of exogenous fibrils. Myr significantly reduced the total amount of cytosolic α-syn fibrils (Figure 1C,D). Since prolonged treatment with fibrils is known to enhance the transcription of α-syn, we also investigated the expression of endogenous α-syn. As shown in Figure 2E, SNCA gene transcription was not modulated by either fibril addition or Myr at the time of treatment. This proves that the reduced α-syn staining is not related to the modulation of protein synthesis but possibly related to its lysosomal degradation.

### 2.2. Myriocin Induces Autophagy and Lysosomal α-Syn Localization in the PD Cell Model

We previously demonstrated that Myr promotes autophagy in response to stress [45]. Here, we demonstrated that the Myr treatment of SH-SY5Y induces α-syn localization in lysosomes, as shown by the co-immunostaining of α-syn and lysosome-associated membrane protein 2 (Lamp2), a lysosomal marker (Figure 2A–G). Interestingly, we observed a more significant co-localization of α-syn inclusions with Lamp2 in Myr-pretreated/fibril-treated cells than in cells exposed to fibrils only (Figure 2C,F), suggesting that autophagy induction by Myr drives α-syn fibrils to lysosomes. Figure 2G shows the quantification of the intensity of the two co-localizing fluorophores according to the intensity correlation quotient (Li’s ICQ).

**Figure 3:**
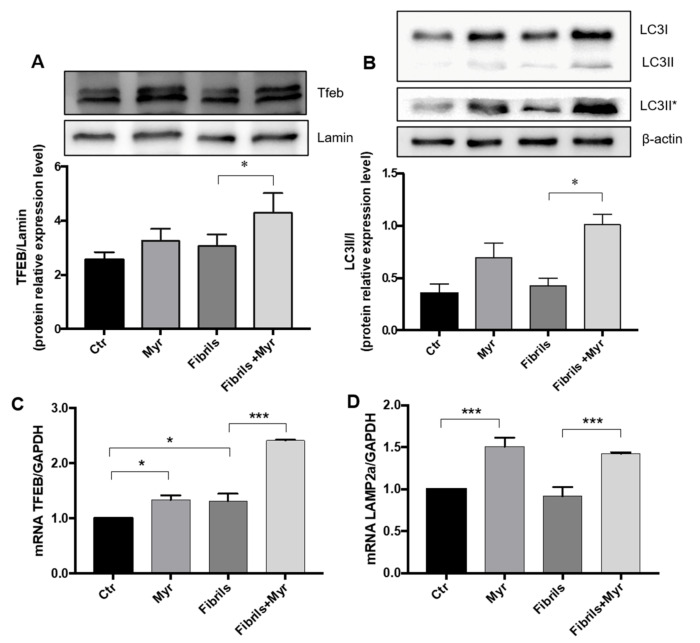
Myr treatment induced TFEB nuclear translocation and autophagy in the PD cell model. Quantification of nuclear translocation of TFEB by Western blot analysis normalized to β-lamin (**A**). Quantification of LC3II/I (**B**) protein expression by Western blot analysis on whole lysates, normalized to β-actin. Protein signals were quantified by densitometric analysis. LC3-II* indicates a longer exposure. Quantification of TFEB (**C**) and LAMP2a (**D**) gene transcription by qRT-PCR in cells treated with 0.5 μM α-syn fibrils for 24 h with or without 50 μM Myr pretreatment. GAPDH was used as a housekeeping gene. All data are expressed as mean ± SEM (* *p* < 0.05; *** *p* < 0.001); one-way ANOVA test followed by Bonferroni correction was used for all data.

TFEB controls lipid metabolism, inflammation, and oxidative stress by inducing autophagy. Here, we observed that Myr activates TFEB by promoting its nuclear translocation in fibril-treated cells (Figure 3A), and this is accompanied by autophagy induction, as indicated by the accumulation of LC3II (Figure 3B). 

Moreover, Myr modulated gene expression in a PD cellular model (Figure 3B), in line with what was previously reported by our group [45]. Myr increased the mRNA expression levels of TFEB and of one of its downstream-regulated genes, LAMP2a, both in cells treated with Myr only and in fibril–Myr-treated cells (Figure 3C,D). 

Moreover, we observed an increase in the levels of LC3-II protein after Myr treatment (Figure 3C). These findings suggest that Myr-induced autophagy is sustained by the upregulation of TFEB and LAMP2a, demonstrating the involvement of autophagy underlying the effect of Myr on reducing cytosolic α-syn fibrils.

### 2.3. Myriocin Modulates Sphingolipids and Reduces Inflammation and Oxidative Stress in PD Cell Model

**Figure 4:**
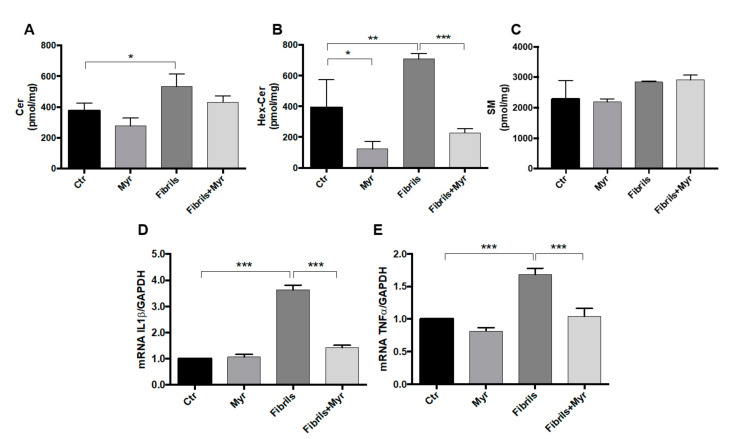
Myriocin reduces α-syn-induced inflammation in SH-SY5Y cells. (**A**–**C**) LC-MS measurement of ceramide (Cer), hexosylceramide (HexCer), and sphingomyelin (SM) levels and Myr effects in SH-SY5Y cells treated with 0.5 μM α-syn fibrils for 24 h with or without 50 μM Myr pretreatment. (**D**,**E**) Quantification of IL1-β and TNFα gene transcription by qRT-PCR in the PD cell model. GAPDH was used as a housekeeping gene. All data, derived from three independent experiments, are expressed as mean ± SEM (* *p* < 0.05; ** *p* < 0.01; *** *p* < 0.001); one-way ANOVA test followed by Bonferroni correction was used for all data.

The fibril treatment of SH-SY5Y cells induced an increase in ceramide and hexosylceramide levels; ceramides were moderately reduced and hexosylceramides significantly reduced in Myr-pretreated cells compared to in fibril-treated cells that were not pretreated (Figure 4A,B). Sphingomyelin (SM) levels were not significantly affected (Figure 4C). Moreover, Myr treatment reduced the fibril-induced upregulation of IL-1β (Figure 4D) and TNFα gene expression (Figure 4E), which are well-known proinflammatory markers. 

Moreover, in order to investigate the effect of Myr on fibril-mediated oxidative stress, we quantified the production of malondialdehyde, an advanced lipo-oxidation end-product implicated in age-related chronic diseases and in PD [46].

**Figure 5:**
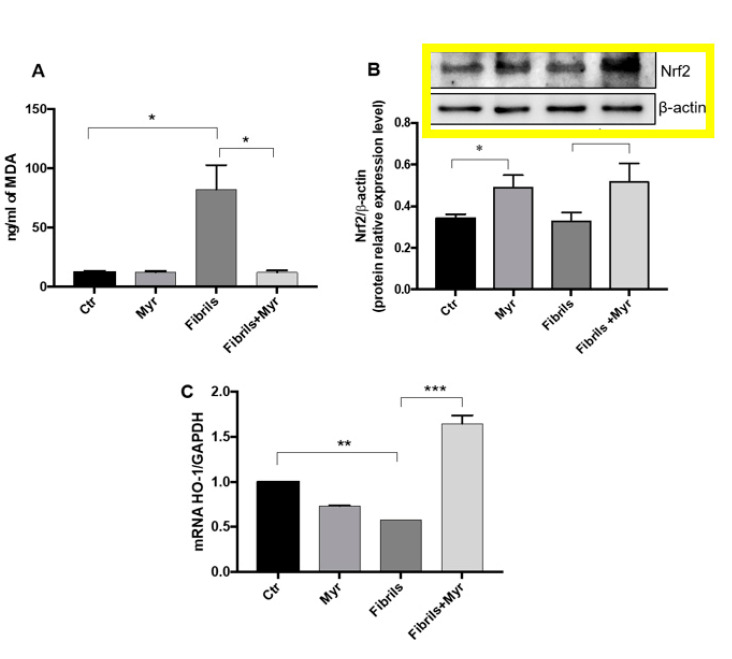
Myr treatment reduces oxidative stress in the PD cell model. (**A**) Measurement of lipid peroxidation in cell supernatant using MDA ELISA kit on SH-SY5Y cells treated with 0.5 μM α-syn fibrils for 24 h with or without 50 μM Myr pretreatment. (**B**) Quantification of Nrf2 protein expression by Western blot analysis on whole lysates and normalized to β-actin. Protein signals were quantified by densitometric analysis. (**C**) Quantification of HO-1 gene expression by qRT-PCR. GAPDH was used as a housekeeping gene. All data, derived from three independent experiments, are expressed as mean ± SEM (* *p* < 0.05; ** *p* < 0.01; *** *p* < 0.001); one-way ANOVA test followed by Bonferroni correction was used for all data.

Myr significantly reduced the oxidative stress induced by the accumulation of α-syn fibrils in treated cells (Figure 5A). Moreover, to understand the mechanism underlying the antioxidant effect of Myr, we evaluated the activation of a key gene involved in the antioxidant response. In particular, we studied nuclear factor erythroid 2-related factor 2 (NRF2) and the expression of one of its activated genes, heme oxygenase 1 (HO-1), in fibril-treated cells after Myr administration.

In homeostatic conditions, NRF2 is constantly degraded via ubiquitination. We evaluated its activation by Western blotting and showed that Myr significantly increased the cytosolic level of the protein (Figure 5B), as expected under sustained autophagy. Similarly, Myr was able to increase the expression of HO-1, which was reduced by fibril treatment (Figure 5C), suggesting that Myr could reduce α-syn-induced oxidative stress by NRF2/HO-1 activation in SH-SY5Y cells. 

### 2.4. Myriocin Restores the Expression of Genes Involved in Intracellular Transport and Synaptic Release in the PD Cellular Model

**Figure 6:**
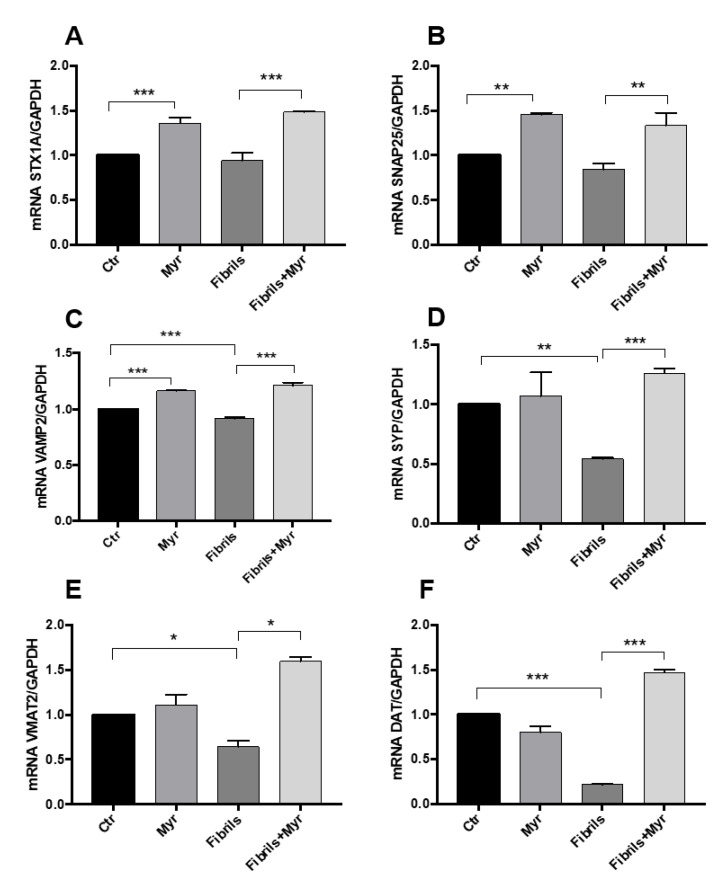
qRT-PCR quantification of genes involved in SNARE complex formation. (**A**–**D**) neurotransmitter vesicle transport, VMAT2 (**E**) and dopamine transporter DAT (**F**). GAPDH was used as a housekeeping gene. All data, derived from three independent experiments, are expressed as mean ± SEM (* *p* < 0.05; ** *p* < 0.01; *** *p* < 0.001); one-way ANOVA test followed by Bonferroni correction was used for all data.

To investigate the effect of Myr on the cellular modulation of neurotransmitter vesicle traffic, we evaluated the expression of genes coding for two SNARE complex proteins—T-SNARE syntaxin 1A (STX1A) and synaptosome-associated protein 25 (SNAP 25)—both of which are located at neuronal synaptic membranes, as well as V-SNARE vesicle-associated membrane protein 2 (VAMP2) and synaptophysin (SYP), which are associated with vesicle membranes [47]. As expected, fibrils significantly reduced the expression of the vesicle-associated VAMP2 proteins (Figure 3C) [47]. As reported in Figure 3A–C, Myr treatment increased the expression of all of the above-mentioned genes, even under fibril treatment. We also observed a significant reduction in the expression of vesicular monoamine transporter 2 (VMAT2), a gene coding for a specific neurotransmitter vesicular transporter, and of DAT, a gene coding for the dopamine transporter; Myr was able to restore theirexpression (Figure 6A–F). 

## 3. Discussion

We here describe the effects of targeting sphingolipid biosynthesis with a specific inhibitor (Myr) to restore the altered lipid metabolism in a cellular model of PD. Exogenously preformed α-syn fibrils are known to be taken up by neuronal cells, causing a proteinopathy-related stress, inducing the formation of LB-like intracellular structures. This triggers oxidative stress and inflammation and impairs cellular functions, mimicking the alterations observed in PD [48]. Sphingolipid synthesis was later shown to be upregulated in postmortem PD brains, with an unbalanced ratio of sphingolipid metabolites [38]. We previously demonstrated that cystic fibrosis and retinitis pigmentosa proteinopathies are responsible for increased sphingolipid synthesis and inflammatory ceramide accumulation and that the inhibition of sphingoid base formation reduces inflammation and cell death [44,45]. Importantly, we demonstrated that Myr reduces not only sphingolipids but also other lipids, such as glycerolipids [45,49,50]. This may occur because the blockade of new sphingolipid synthesis is sensed as an alert signal by the cell. Indeed, we showed that Myr promotes a TFEB-induced stress response. TFEB initiates a transcriptional program that reduces inflammation and oxidative stress, induces autophagy [45,51], organizes cellular lipids by increasing their oxidation [45,51,52] but also by favoring lipid storage formation [8]. These activities all contribute to reducing most of the cellular lipid species [45]. Upon fibril treatment, α-syn inclusions in neuronal cells are effectively reduced by Myr via an increase in their co-localization in the lysosomal compartment. This is explained by the Myr-induced promotion of TFEB-mediated autophagy. Myr significantly induces TFEB nuclear translocation in fibril-treated cells, thus strengthening cellular stress defenses. Although fibril treatment does not suppress TFEB and LAMP2 transcription, Myr significantly activates it, as expected from our previous studies [45]. Such a transcriptional program acts to increase autophagy, as shown by LC3II accumulation, thus directing α-syn to lysosomes for degradation. The increased transcription of not only TFEB but also its downstream transcriptional target and lysosomal gene, LAMP2, demonstrates the known action of TFEB in promoting lysosomal genesis, reducing cell stress. 

Myr counteracts the fibril-induced increase in ceramide and significantly lowers the increase in hexosylceramides. Hexosylceramide accumulation is linked to defective ER, Golgi traffic, and lysosomal activities associated with oxidative stress, impaired autophagy, and neurodegeneration [53,54,55]. As expected, Myr reduces the transcription of inflammatory mediators and almost abolishes the profound effect on lipid peroxidation of fibril treatment. Oxidative stress is a major cause of PD progression. The pharmacological activation of NRF2, a key transcriptional factor that sustains the response to oxidative stress, is considered a therapeutic strategy for the prevention of PD neurodegeneration [23,25]. Myr’s antioxidant action is exerted via inducing NRF2 transcriptional activity. PD is characterized by the loss of dopamine signaling due to the dysfunction of α-syn. The latter regulates synaptic functions and plasticity, interacting with the SNARE proteins that mediate the docking of neurotransmitter vesicles, exocytosis, and neurotransmitter release [56]. Thus, PD neurodegeneration initiates as a synaptopathy [57], and postmortem PD brains exhibit an altered balance of the SNARE proteins [58], which are sequestered within LBs [57]., The progression of degeneration leads to the reduced expression of genes involved in SNARE complex formation [59]. Upon treatment with fibrils, we observed a reduced expression of genes coding for SNARE complex (SYP and VAMP2) proteins, for the monoamine SLC vesicular transporters VMAT2, and for the dopamine transporter DAT. It was also observed that protecting cells against fibril stress by Myr co-treatment significantly increased the transcription of all of the above-mentioned genes, indicating that Myr transcriptionally favors the recovery of cellular functions. In addition, we noted a significant Myr-induced increase in other SNARE genes, such as SNAP25 and STX1A, suggesting that the inhibition of sphingolipid synthesis transcriptionally regulates the vesicular transport of neuromediators. 

This study has strengths and limitations. The use of exogenously preformed α-syn fibrils quickly induces an intracellular overload, which we observed within 24 h, whereas PD is a chronic and progressively accumulating form of stress; thus, the response may differ under sustained and prolonged stimulation, especially if transcriptional activities are involved. Moreover, SH-SY5Y cells are not differentiated neurons and do not form synapses or release dopamine. Nonetheless, these cells can model the PD alteration of vesicle traffic because they express all of the genes involved in dopamine formation and release [60]. The strength of our study lies in the demonstration that α-syn-induced proteinopathy can be counteracted by promoting a metabolic shift that is driven by slowing down sphingolipid synthesis, thereby transcriptionally modifying the PD phenotype.

## 4. Materials and Methods

### 4.1. Reagents and Antibodies

For the experiments, we used the following materials: culture media (Gibco-Thermo Fisher Scientific, Waltham, MA, USA); fetal bovine serum and minimum essential medium with Earle’s salt (EuroClone Life Science, Pero, MI, Italy); penicillin/streptomycin and RIPA buffer (Sigma-Aldrich, St. Louis, MO, USA); a protease inhibitor cocktail (Roche, Basilea, Switzerland); Quick Start™ Bradford Dye Reagent and Clarity™ Western ECL Blotting Substrates; iScript^TM^ cDNA synthesis, retro-transcription kit (BioRad, Segrate, MI, Italy); ReliaPrep™ Miniprep RNA Extraction System and GoTaq qPCR Master Mix (Promega, Madison, WI, USA); SYBR Green (Takara, Kyoto, Japan); and synthetic oligonucleotides from Eurofins Genomics (Edersberg, Germany). The primary antibodies used were as follows: anti-Nrf2 (ElabScience, Houston, TX, USA), anti-β-Actin (Sigma-Aldrich, St. Louis, MO, USA), anti-Tfeb (ab2636, Abcam, Cambridge, UK), anti-α-synuclein (ab138501 Abcam, Cambridge, UK), and anti-LAMP2 (Santa Cruz Biotechnology, Dallas, TX, USA). The secondary antibodies were purchased from Jackson Laboratories (Bar Harbor, ME, USA). The secondary antibodies for immunofluorescence were as follows: Alexa Fluor 488 and Alexa Fluor 546; these were purchased from Thermo Fisher, and the Human MDA (Malondialdehyde) ELISA Kit was purchased from Fine Test, Labospace (Milan, MI, Italy).

### 4.2. Cells and Treatments

SH-SY5Y cells, a human neuroblastoma cell line provided by ATCC, were grown in DMEM supplemented with 10% FBS and 1% penicillin/streptomycin at 37 °C and 5% CO_2_. Pretreatment with Myr (50 µM) was performed for 1 h in 100 mm dishes seeded with 1 × 10^5^ cells each. When indicated, cells were exposed to 0.5 µM preformed α-syn fibrils (PFFs) for 24 h. Samples were prepared at least in triplicate for each experiment, except for those differently indicated.

### 4.3. Preparation of Preformed α-Syn Fibrils 

LPS-free recombinant α-syn was produced and purified as previously described, and α-syn fibrils were obtained by aggregating monomeric α-syn for 7 days at 37 °C with shaking at 1000 rpm (ThermoMixer F1.5 Eppendorf, 1000 rpm) [61]. α-Syn monomer was resuspended in sterile PBS at 280 µM for the aggregation assay, and after 7 days, fibrils were collected by centrifugation to remove residual soluble monomeric or oligomeric species in the supernatant. α-Syn fibrils were then resuspended to a final concentration of 200–250 µM in sterile DBPS, sonicated with a power input of 20 to 30 W for 30 s (pulsing on/off) at 4 °C (Covaris S2 Ultrasonicator) and stored at −70 °C until use.

Quality control was performed using transmission electron microscopy (TEM). Briefly, preformed fibrils were diluted 1:3 in Milli-Q H_2_O, deposited on a copper grid, washed twice, and directly stained with 2% uranyl acetate. Samples were then observed via TEM (FEI Tecnaii G2) [48].

### 4.4. Lipid Analysis

The lipids were extracted from cell pellets (200 µg of protein) by monophasic extraction with water:chloroform:methanol (1:3:6, *v*/*v*/*v*) coupled to alkaline methanolysis. The purified extracts were analyzed by LC Dionex 3000 UltiMate (ThermoFisher Scientific, Waltham, MA, USA) coupled to a tandem mass spectrometer AB Sciex 3200 QTRAP (Sciex, ON, CA). The separation was achieved by reversed-phase chromatography using BEH C8 100 × 2.1 mm × 1.7 μm (Waters, Milford, MA, USA) with a linear gradient between eluent A (0.2% formic acid and 2 mM ammonium formate water solution) and eluent B (methanol, 0.2% formic acid, and 1 mM ammonium formate) [62]. Mass spectrometry measurement was conducted by multiple reaction monitoring (MRM). The product ion *m/z* 264 (corresponding to sphingosine) was examined for each precursor ion of ceramides and hexosylceramides, whereas for sphingomyelins, the product ion *m/z* 184 (corresponding to the choline head) was examined. Specifically, the analysis comprised the sphingolipids, ceramides (Cer, DP 40 eV, and CE 35 eV), hexosylceramides (HexCer, DP 40 eV, and CE 50 eV), and sphingomyelins (SM, DP 40 eV, and CE 50 eV) with fatty acids from C16 to C24. Quantitative analysis was performed using the MultiQuant software (ver 1.2, Sciex, ON, CA).

### 4.5. Microscopy Analysis

Subconfluent SH-SY5Y cells were fixed in 4% paraformaldehyde, permeabilized in 0.1% Triton X-100, and blocked in 1% BSA–PBS for 2 h. Then, the cells were incubated in PBS buffer with primary antibodies against human α-synuclein and human LAMP2 overnight at 4 °C and washed twice in PBS followed by incubation with secondary antibodies: Alexa Fluor 555 for α-synuclein immunofluorescence and Alexa Fluor 488 and Alexa Fluor 546 for co-immunofluorescence (1:100). The nuclei were stained with DAPI (1:1000 dilution) [63]. For negative controls, we incubated the cells with secondary antibody only, to exclude nonspecific binding. Confocal images were acquired using a Nikon A1 Laser Scanning Confocal Microscope (60x oil immersion objective). Image quantitation was performed using the Fiji or ImageJ analysis software. Fluorescence co-localization (the correlation index) was determined by evaluating fields acquired blindly in each group and by applying the same selection strategy. The images were analyzed by an automated Fiji–ImageJ plugin, JACoP [64], to determine Li’s ICA of isolated cells labeled as the region of interest and excluding clustered cells. 

### 4.6. ELISA Kit

The levels of oxidative stress were measured using an MDA ELISA kit (Fine Test, Labospace, Milan, MI, Italy) following the manufacturer’s protocols. Briefly, SH-SY5Y cells were treated with or without Myr (50 µM) for 24 h. Following the treatments, the supernatant was collected and centrifuged at 1000× *g* for 20 min at 2–8 °C to remove insoluble impurities and cell debris. The supernatant was dispensed into the wells; 50 μL of biotin-labeled antibody (included in the kits) was added, and the plate was incubated at 37 °C for 45 min. The samples were washed three times with wash buffer before adding 100 μL of HRP–streptavidin conjugate (SABC). The plate was incubated at 37 °C for 30 min. The wells were washed three times with washing buffer followed by the addition of 90 μL of TMB (3,3′,5,5′-tetramethylbenzidine) substrate solution; the plate was incubated at 37 °C in the dark for 15–20 min. After adding the stop solution into each well, the absorbance at 450 nm was measured using a microplate reader (EnSight™, PerkinElmer, Waltham, MA, USA).

### 4.7. Protein Extraction and Western Blotting

For the Western blots for transcriptional factors, nuclear and cytoplasmic extracts were obtained from cells with the NE-PER Nuclear and Cytoplasmic Extraction Reagents kit (ThermoFisher Scientific) according to the manufacturer’s instructions. Total cellular proteins were extracted from cells in RIPA buffer. The concentrations of proteins in the lysates were measured using Quick Start™ Bradford Dye Reagent (reading the OD at 595 nm). Then, 20 μg of proteins per sample were separated on SDS-PAGE gels and electroblotted onto nitrocellulose (for NRF2) and PVDF (for LC3) membranes. After washing in Tris-buffered saline containing 0.1% Tween-20 (TBS-T) and blocking with 5% non-fat dry milk for 1 h at room temperature, the membranes were probed overnight at 4 °C with the primary antibodies. The morning after, the membranes were washed three times in TBS-T and then incubated with the horseradish peroxidase-conjugated secondary antibodies [65]. After the final washes, proteins were detected using an enhanced chemiluminescent horseradish peroxidase substrate, and the relative band intensities were captured and quantified using Alliance™ UVITEC Cambridge (UK). For LC3 Western blotting, the images of LC3I and LC3II were detected at different exposure times for the chemiluminescence detector as previously indicated [66].

### 4.8. RNA Extraction and qRT-PCR

Total RNA was isolated from harvested cells with the ReliaPrep™ Miniprep RNA extraction system (Promega, Madison, WI, USA), according to the manufacturer’s instructions. 

Then, 1 µg of purified RNA was reverse transcribed to cDNA. The amplification was performed for the following target genes: α-SYN, LAMP2A, HO-1, IL-1β, TNF-α, TFEB, STX1A, VAMP2, SNAP25, SYP, DAT, and VMAT2; all the sequences are available on request. The relative mRNA expression of the target genes was normalized to the endogenous GAPDH control gene and is represented as the fold change versus control, calculated by the comparative CT method (∆∆CT method). The analysis was performed by referring to control values that did not significantly differ from one another (triplicate samples; their standard deviation divided by their mean was <1) [27]. Real-time PCR was performed using SYBR Premix Ex Taq™ II (Takara, Kyoto, Japan); all the other sequences are available on request. 

### 4.9. Statistical Analysis

The data are expressed as mean ± SEM and were calculated from experimental replicates. Significance was evaluated by one-way ANOVA tests followed by Bonferroni correction (*p* < 0.05) or two-tailed Student’s *t*-tests, as specified in the figure legends. Statistical analysis was performed using the GraphPad Instat software, and the graph illustrations were generated by the GraphPad Prism software version 8 for MacOs (La Jolla, CA, USA).

## 5. Conclusions

**Scheme 1:**
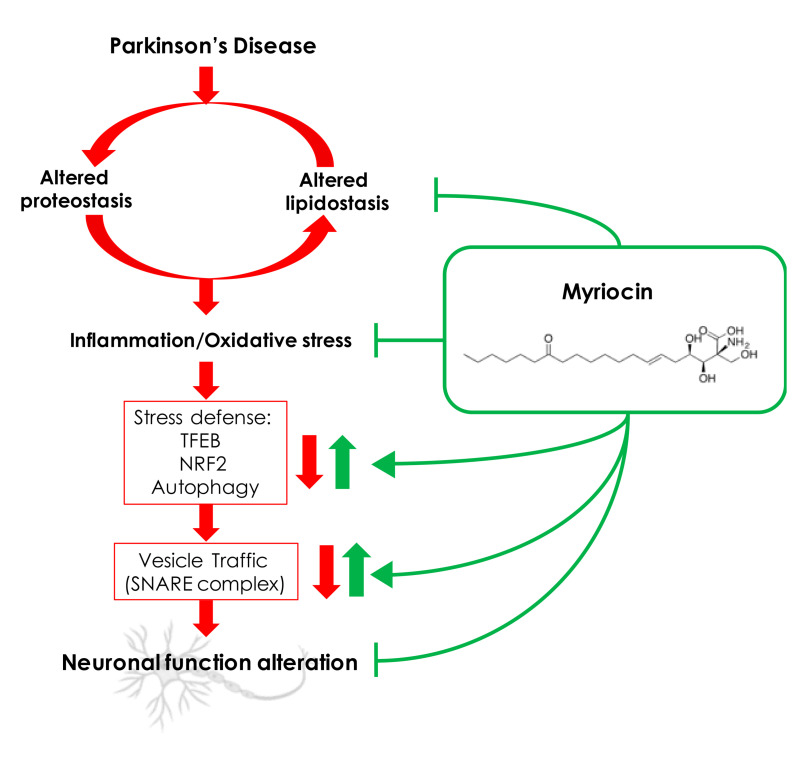
Proposed therapeutic action of Myr in the PD cellular model.

PD is a proteinopathy associated with defective intracellular vesicle traffic and altered lipid homeostasis (Scheme 1). The inhibition of de novo sphingolipid synthesis activates a transcriptional response, involving autophagy and an antioxidant defense mechanism, that restores the expression of the proteins required for neurotransmitter vesicle traffic. The regulation of lipid synthesis and metabolism may be an innovative and promising strategy for counteracting PD neurodegeneration. 

## Data Availability

The data presented in this study are available on request from the corresponding author.

## Abbreviations

ASAH1	N-acylsphingosine amidohydrolase 1
α-syn	α-synuclein
ATP6VOA1	ATPase H+ transporting V0 subunit a1
Cer	ceramide
CTS	cathepsin
DGKQ	diacylglycerol Kinase theta
GALC	galactosylceramidase
GBA	glucosylceramidase beta
HO-1	heme oxygenase 1
ICQ	intensity correlation quotient
LB	Lewy bodies
LRRK2	leucine-rich repeat kinase 2
Myr	myriocin
NRF2	nuclear factor (erythroid-derived 2)-like 2
nSMase	neutral sphingomyelinase
PANK2	pantothenate kinase 2
PD	Parkinson’s disease
PLA2G6	phospholipase A2 group VI
PPARs	peroxisome proliferator activated receptors
SYNJ1	synaptojanin 1
SNAP25	synaptosomal-associated protein 25
SNARE	soluble N-ethylmaleimide-sensitive factor attachment receptor
SM	sphingomyelin
SMPD1	sphingomyelin phosphodiesterase 1
STX1A	syntaxin 1A
SREBF1	sterol regulatory element binding transcription factor 1
TFEB	transcription factor EB
VAMP2	vesicle-associated membrane protein 2
VMAT2	vesicular monoamine transporter 2
VPS35	VPS35 retromer complex component
